# Supplementary figures and images for: Deep mining of the Sequence Read Archive reveals major genetic innovations in coronaviruses and other nidoviruses of aquatic vertebrates
Source: PLoS Pathog. 2024 Apr 22;20(4):e1012163. doi: 10.1371/journal.ppat.1012163 (PMC11065284; doi:10.1371/journal.ppat.1012163)

## score decile

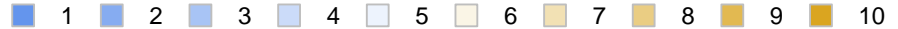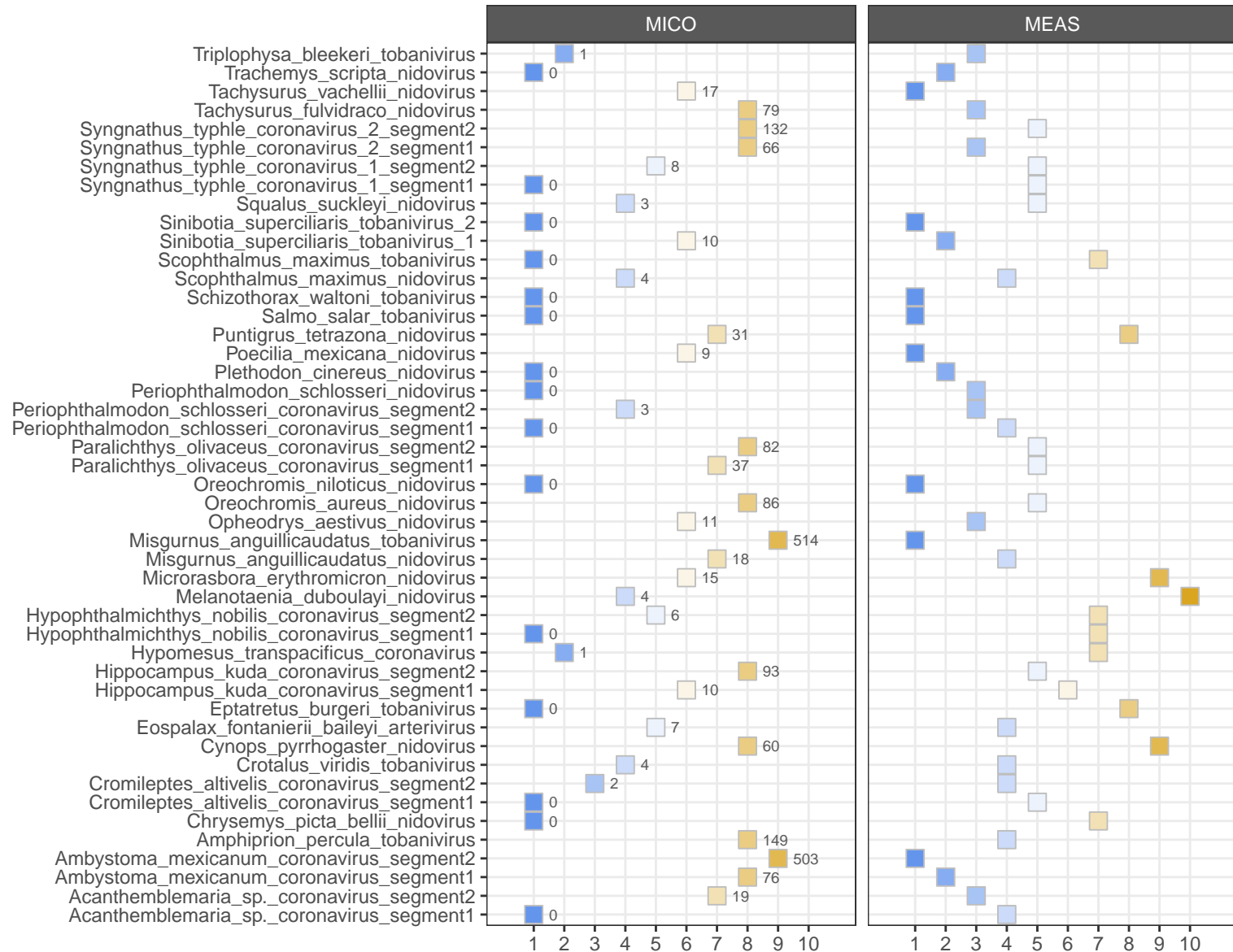

Supplement: S1 Fig — The continuous meas and mico values calculated for each novel nidovirus sequence were mapped to deciles of the meas and mico distributions of a reference set consisting of 2350 RNA virus sequences to obtain MEAS (left) and MICO (right) metrics. The numbers next to the MICO symbols indicate the original mico value, e.g. the minimum read coverage observed for the contig across its entire length excluding the terminal 100 nt at both ends. (PDF) [file ppat.1012163.s001.pdf]

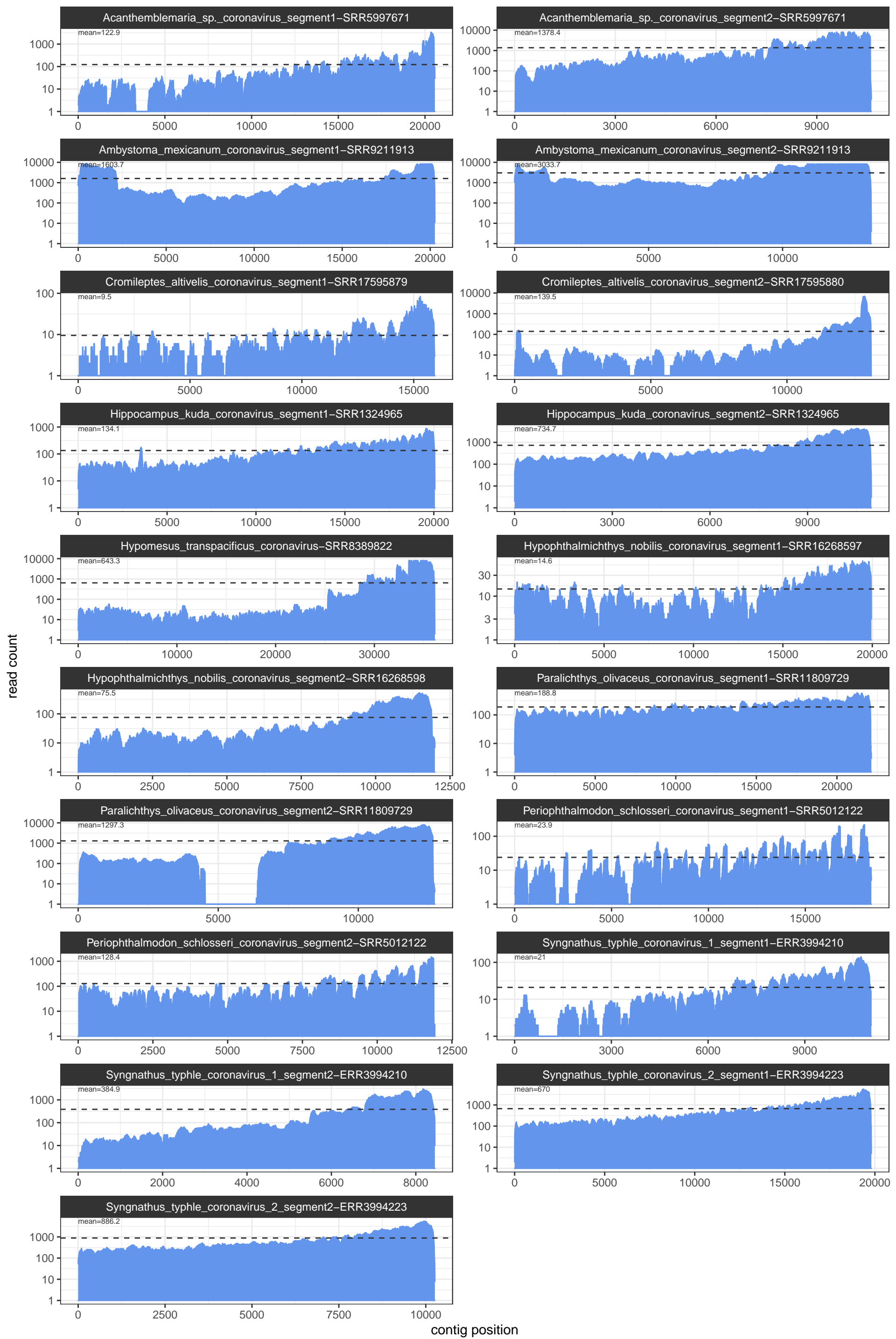

Supplement: S2 Fig — Mean coverage value is indicated and highlighted by the horizontal dashed line. (PDF) [file ppat.1012163.s002.pdf]

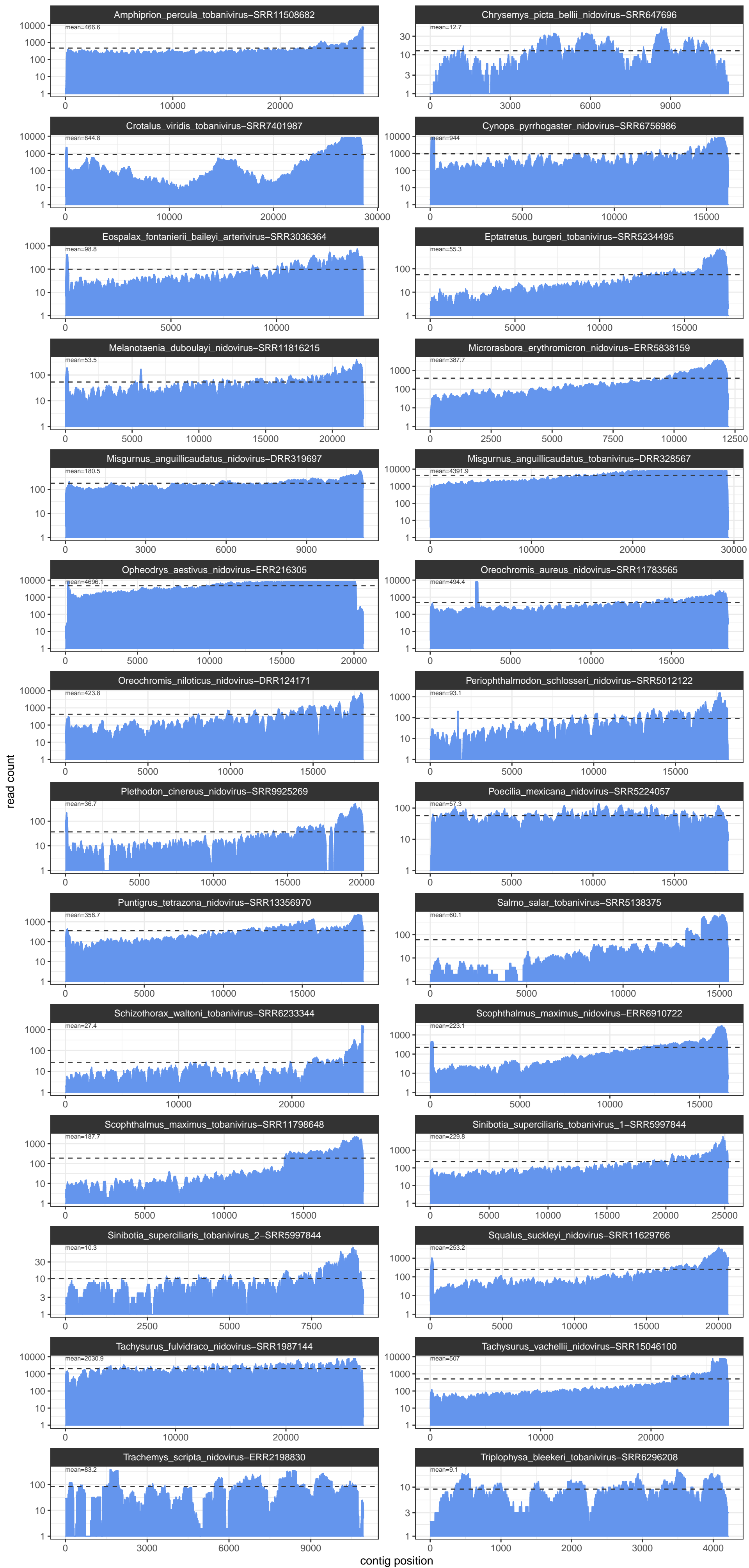

Supplement: S3 Fig — Mean coverage value is indicated and highlighted by the horizontal dashed line. (PDF) [file ppat.1012163.s003.pdf]

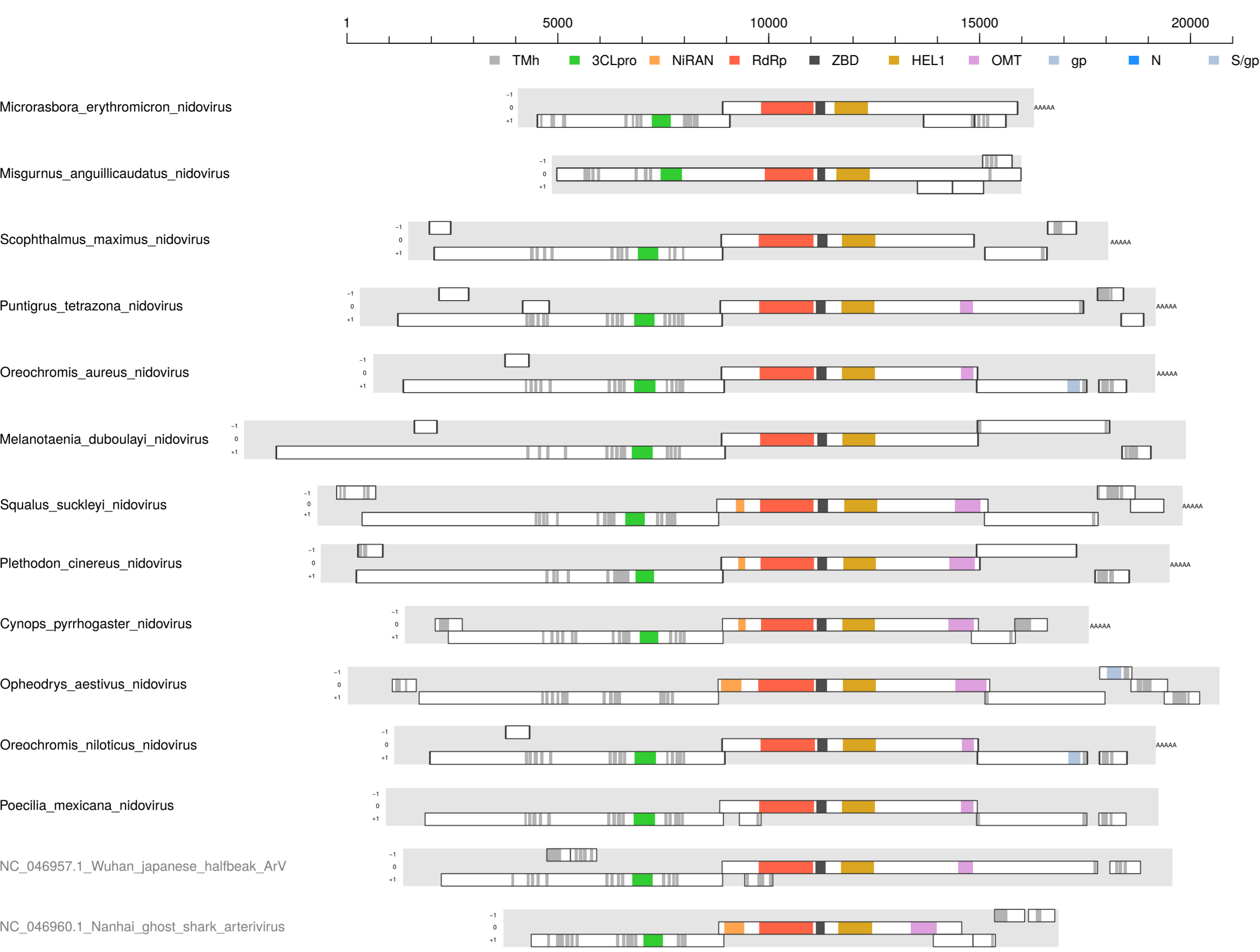

nanhypo/nangosha-like

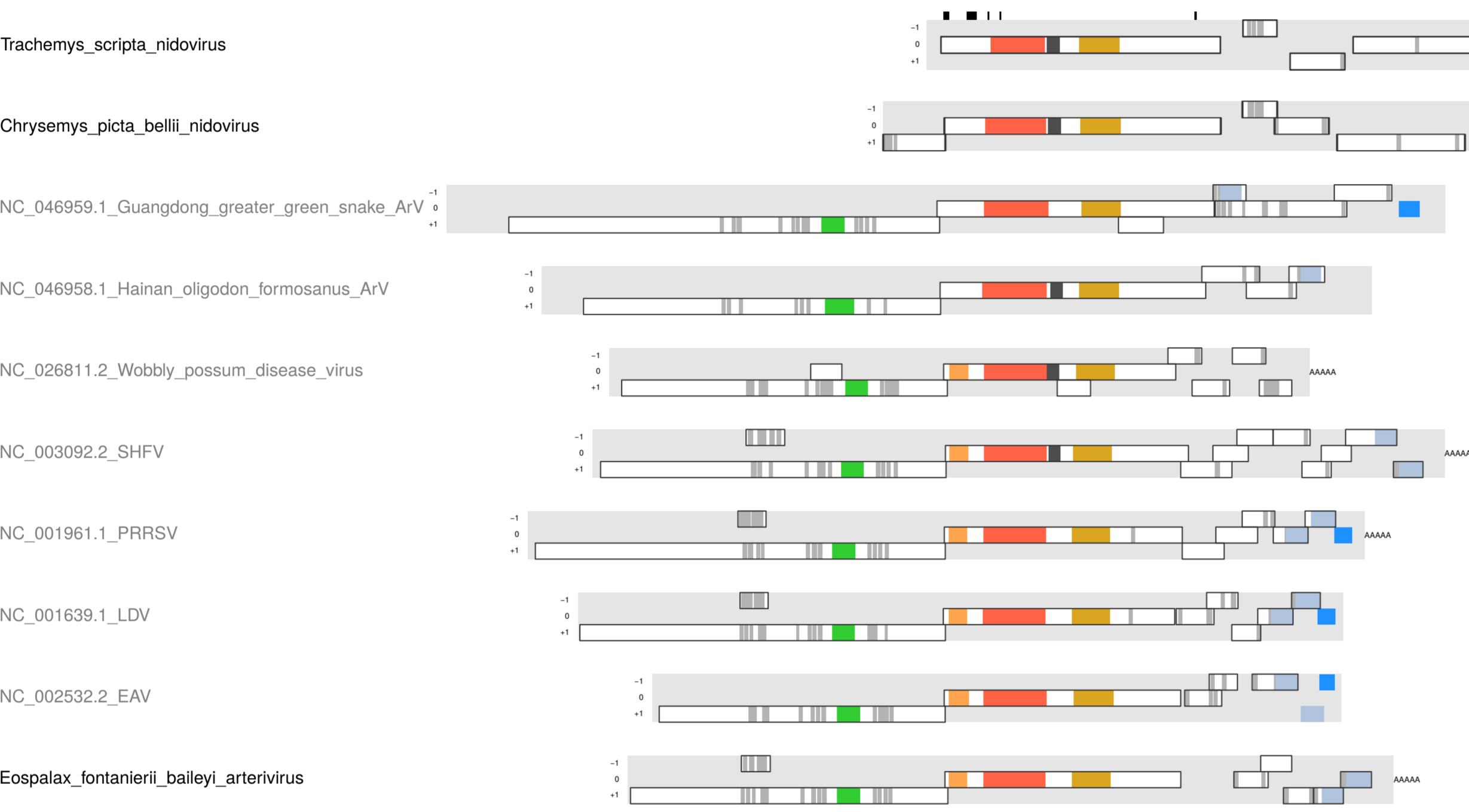

arteri-like

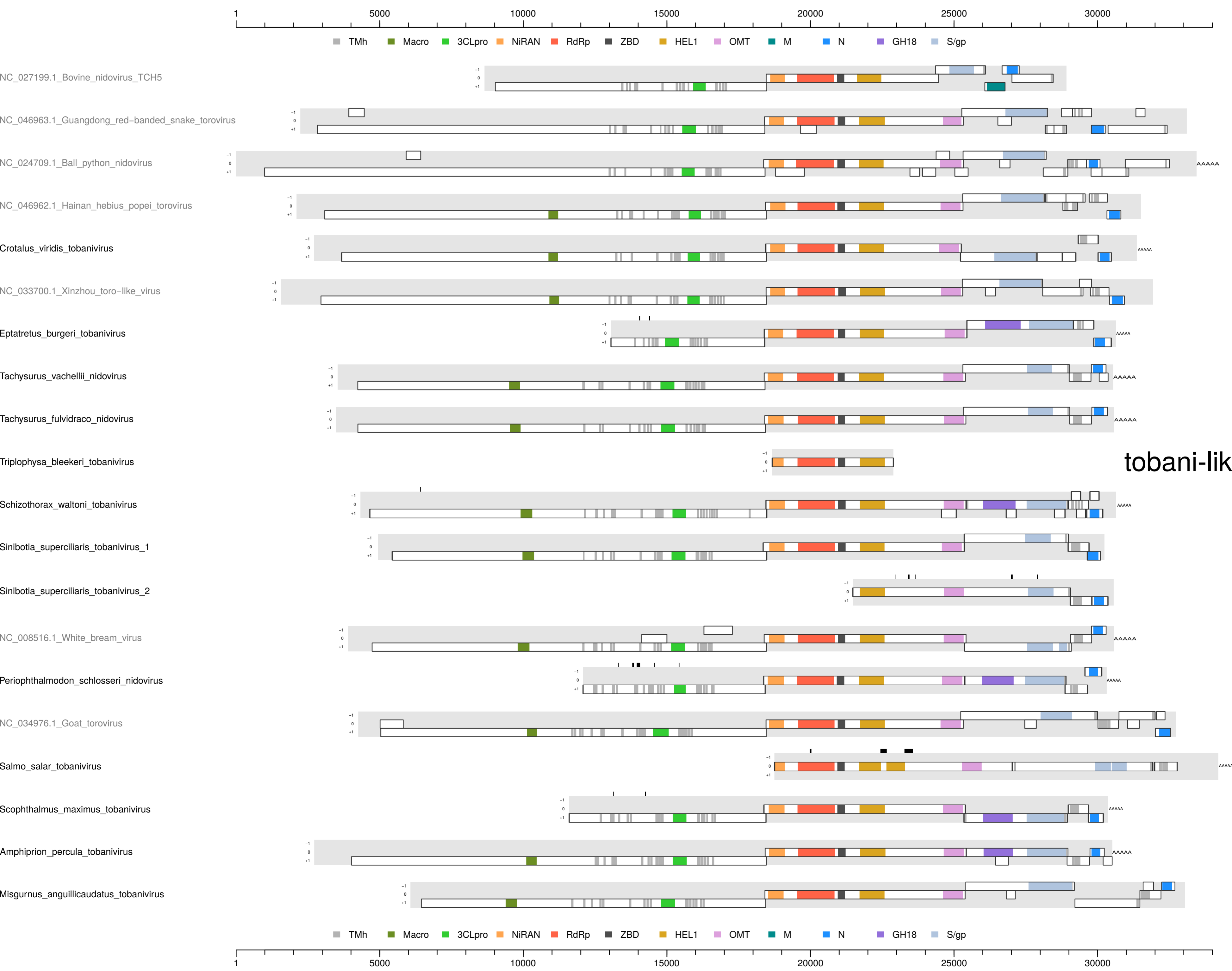

tobani-like

Supplement: S4 Fig — Names of newly viruses discovered in this study are in black, those of reference viruses in gray. Predicted open reading frames (ORFs) of at least 300 nucleotides in length are shown as white rectangles; ORFs are defined to start and end at a stop codon. Protein domains predicted via profile HMM are indicated in color. See legend of main Fig 4 for further details. (PDF) [file ppat.1012163.s004.pdf]

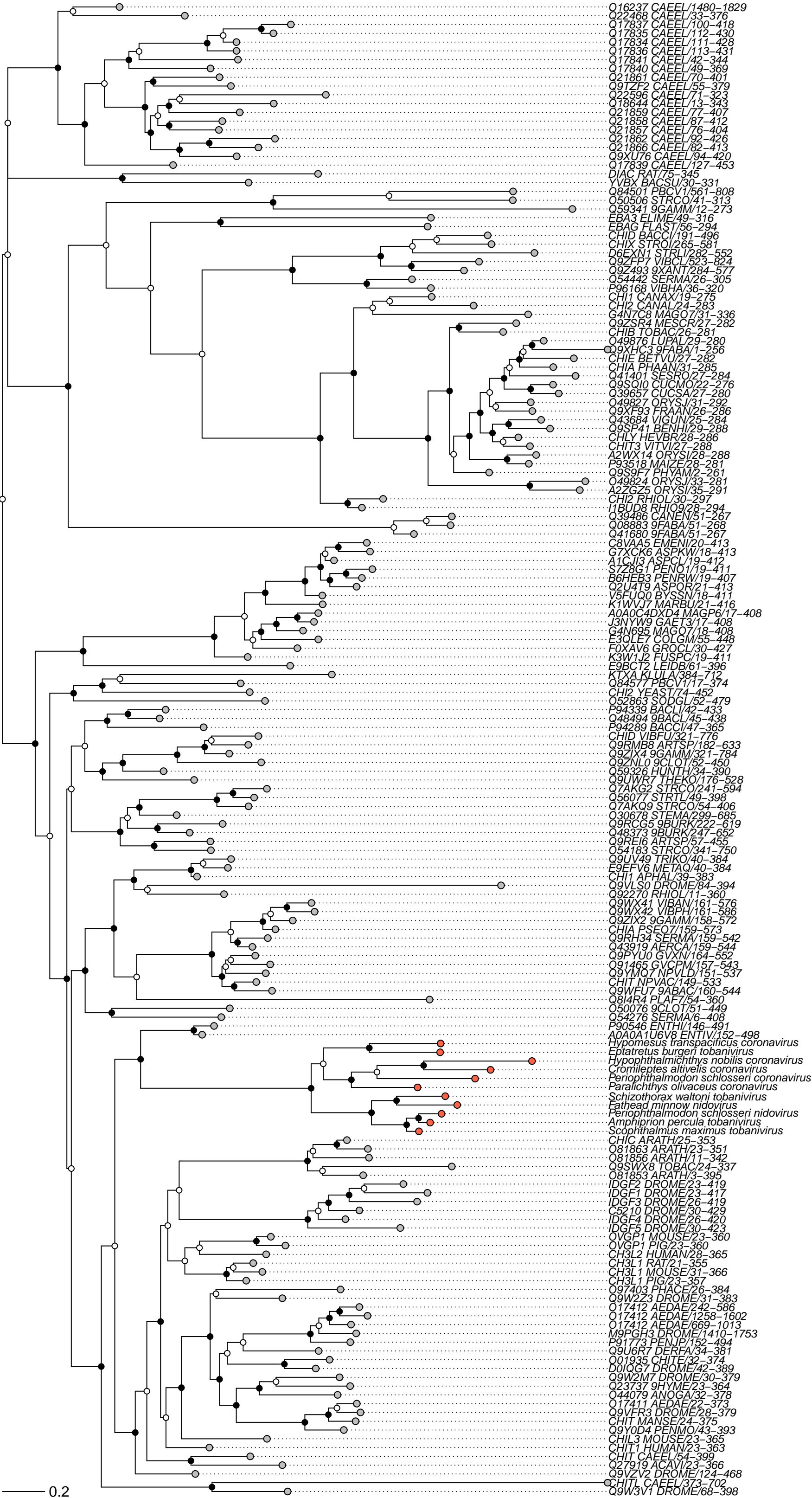

Supplement: S6 Fig — Tips corresponding to nidoviruses are highlighted using red circles; gray circles otherwise. Tip labels start with UniProt accessions in the case of cellular proteins and details about sequences such as host information can be obtained via www.uniprot.org. White and black circles at internal nodes indicate SH-like branching support smaller and larger than 0.8, respectively. The branch lengths are in units of aa substitutions per site; scale bar is shown. (PDF) [file ppat.1012163.s006.pdf]
