# Supplementary material for: Deep mining of the Sequence Read Archive reveals major genetic innovations in coronaviruses and other nidoviruses of aquatic vertebrates
Source: PLoS Pathog. 2024 Apr 22;20(4):e1012163. doi: 10.1371/journal.ppat.1012163 (PMC11065284; doi:10.1371/journal.ppat.1012163)

A

	3‘-RACE PCR			
**Virus**	**Oligo**	**Sequence**	**Tm**	**bp**
StyCoV-1 Segment 1	3-V1S1-out	ACACATTCCGCCAGTGCTAT	57	~1040
	3-V1S1-in	TGCAAGTGGGTCTCTTCTCG	57	~880
StyCoV-1 Segment 2	3-V1S2-out	CACGTAAAGCACGTTCGGTC	57	~920
	3-V1S2-in	ACCTGAAGTGGATGCCCTGT	58	~600
StyCoV-2 Segment 1	3-V2S1-out	TAGTGGCTTGGATCGTTCCG	57	~870
	3-V2S1-in	GCCCCGGTGCATTTAACGTA	58	~520
StyCoV-2 Segment 2	3-V2S2-out	AACTTCGATGCTTTGGCTGC	57	~840
	3-V2S2-in	AAACCACCCCGTAGTGTTCG	57	~590

C

		Overgap PCR			
		**Oligo**	**Sequence**	**Tm**	**bp**
cDNA synthesis		r5-V2S2-#02	GGTGCTCTTTGGCAACTCAC	57	
		r3-V2S1-out	AGATTCCGCGTACACTACGC	57	
5‘ control PCR	Outer	f5-V2S2-#01	GCAACTGCCTTCGTCTAGCA	58	491
		r5-V2S2-#02	GGTGCTCTTTGGCAACTCAC	57	
	Inner	f5-V2S2-#02	GCCCACAGTGAATCTGAGGT	57	147
		r5-V2S2-#01	AACGAGCGAAGCAAAACTGC	57	
3’control PCR	Outer	3-V2S1-out	TAGTGGCTTGGATCGTTCCG	57	811
		r3-V2S1-out	AGATTCCGCGTACACTACGC	57	
	Inner	3-V2S1-in	GCCCCGGTGCATTTAACGTA	58	418
		r3-V2S1-in	AAGGAGATCGAACCGGCAAC	57	
Overgap PCR	Outer	3-V2S1-out	TAGTGGCTTGGATCGTTCCG	57	~1360
		r5-V2S2-#02	GGTGCTCTTTGGCAACTCAC	57	
	Inner	3-V2S1-in	GCCCCGGTGCATTTAACGTA	58	~890
		r5-V2S2-#01	AACGAGCGAAGCAAAACTGC	57	

D

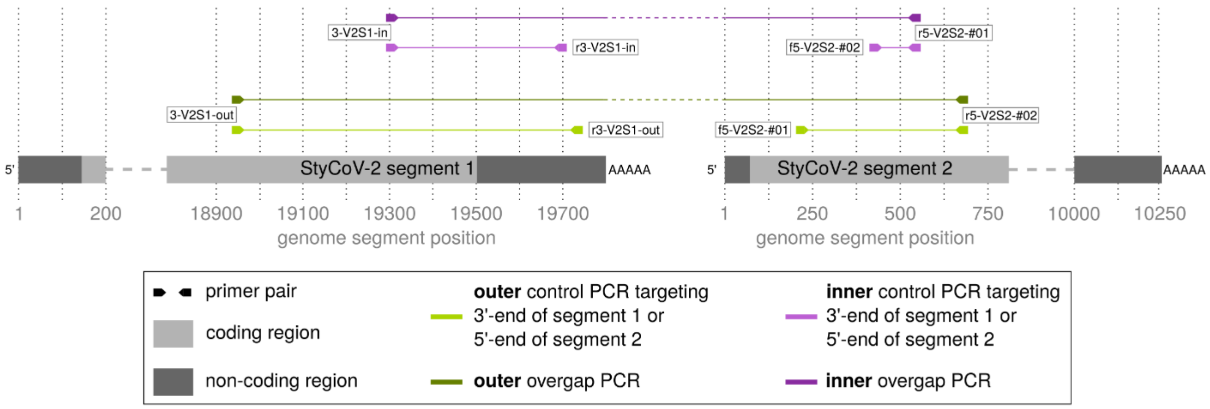

B

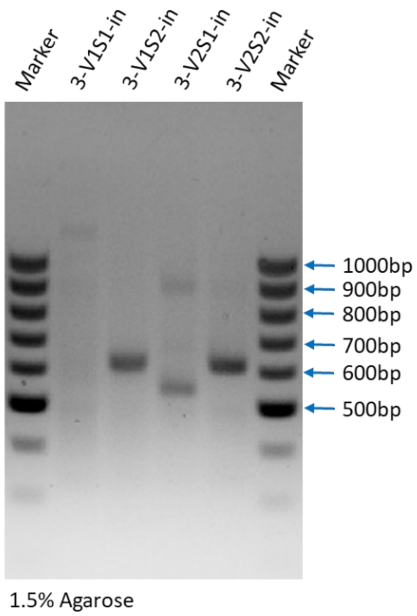

E

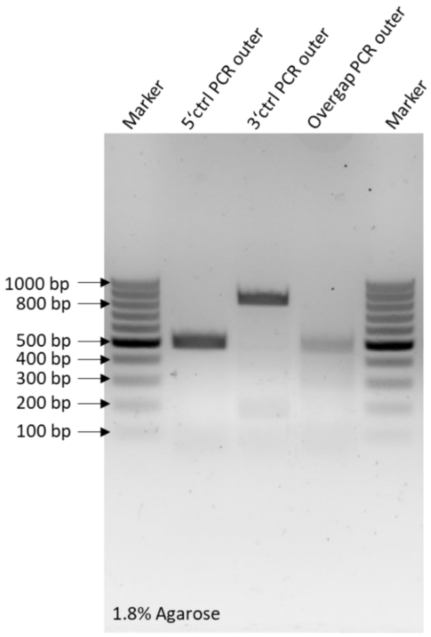

F

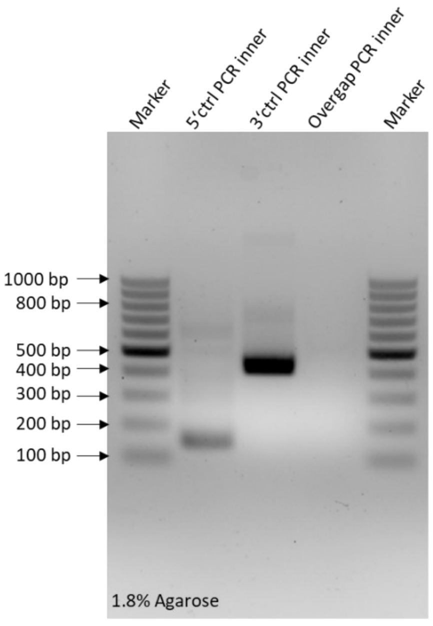

Supplement: S5 Fig — Primers used for 3’RACE PCR (A) and overgap PCR (C), genomic locations of nested primer pairs used for overgap PCR (D) and respective DNA electrophoresis of inner 3’RACE PCR (B), outer overgap PCR (E) or inner overgap PCR (F). Purified PCR products (200 ng) for each segment were loaded and resolved in 1.5% or 1.8% agarose gels. (PDF) [file ppat.1012163.s005.pdf]
