# Supplementary material for: Deep mining of the Sequence Read Archive reveals major genetic innovations in coronaviruses and other nidoviruses of aquatic vertebrates
Source: PLoS Pathog. 2024 Apr 22;20(4):e1012163. doi: 10.1371/journal.ppat.1012163 (PMC11065284; doi:10.1371/journal.ppat.1012163)
Supplement: Attachment — Submitted filename: PPATH_revision1_response_to_reviewer_comments_vFinal.docx [file ppat.1012163.s010.docx]

*Response to reviewer comments to* PPATHOGENS-D-21-02011 -“Deep mining of the Sequence Read Archive reveals bipartite coronavirus genomes and inter-family Spike glycoprotein recombination”

**Deep mining of the Sequence Read Archive reveals major genetic innovations in coronaviruses and other nidoviruses of aquatic vertebrates**

Chris Lauber, Xiaoyu Zhang, Josef Vaas, Franziska Klingler, Pascal Mutz, Arseny Dubin, Thomas Pietschmann, Olivia Roth, Benjamin W. Neuman, Alexander E. Gorbalenya, Ralf Bartenschlager, Stefan Seitz

Below we provide

1) a summary of advances of our study,
2) general notes about advances that address the main concerns and
3) a detailed point-by-point response to the editorial requests and the comments made by three reviewers to PPATHOGENS-D-21-02011.

**1) Summary of advances**

- independent discovery of two fish coronaviruses with bisegmented genomes in unpublished RNA samples of 69 infected fishes
- validation of poly-A tails at both segments of two fish coronaviruses with bisegmented genomes by 3’RACE PCR and subsequent Sanger sequencing
- additional computational analyses supporting genome bisegmentation in a monophyletic lineage of in total 12 coronaviruses
- update of our SRA screen, resulting in the discovery of 14 additional nidoviruses from vertebrate samples
- extra quality control of our assembled nidovirus contigs by a human expert in viral genome assembly
- evidence for discontinuous transcription to produce subgenomic RNAs in two of the discovered nidoviruses
- Advanced computational tools enabling virus genome assembling by deep mining, including Virushunter and Virusgatherer, are placed on the GitHub;
- Independent validation of the discovery of several coronaviruses in published deep-mining research by others

**2) General notes**

We have conducted extensive additional analyses, both experimental and computational, that concern multiple points raised by several of the reviewers and which are therefore briefly summarized in the beginning.

**G1**: We have teamed-up with the authors of the SRA data (the team of Dr. Olivia Roth; now co-authors of this manuscript) in which we identified two of the fish coronaviruses with bisegmented genomes, Syngnathus typhle coronavirus 1 (StyCoV-1) and Syngnathus typhle coronavirus 2 (StyCoV-2). They provided us with unpublished RNA samples and transcriptome sequencing data of 202 fishes of which 69 samples were positive for StyCoV-1 or StyCoV-2. Viral genome assembly of these new 69 NGS datasets were in full agreement with our original findings that the non-structural (segment 1) and structural (segment 2) proteins were encoded on separate contigs. Moreover, we performed a 3’RACE-PCR on the available RNA samples from the infected fishes to determine the sequence of the 3’-termini of the StyCoV-1 and StyCoV-2 genomes. This analysis confirmed the presence of poly-A tails at both segments of two viruses (new main and supplementary illustrations **Fig. 5 and Fig. S5**).

**G2:** To provide additional support for genome bisegmentation of StyCoV-1 and StyCoV-2, we tested a hypothesis that the observed bisegmentation is fortuitous due to missing reads connecting two segments. For each coronavirus we chose the SRA experiment with the highest viral sequencing depth and mapped all reads simultaneously against both segment sequences. We then looked for read pairs that could be expected if the observed segmentation was of the technical origin. They would fulfill the following requirements: (i) The reads of a pair align to different segments in the orientation that might be expected if bisegmentation is due to missing sequence that would join the two segments into a single sequence, (ii) One read of a pair aligns in proximity to the 3’-end of segment 1 (or 5’-end of antisense segment 1) and the other read aligns in proximity to the 5’-end of segment 2 (or 3’-end of antisense segment 2), with proximity defined by the expected insert size (plus some random variance) of a sequencing data set, (iii) Both reads of a pair show reasonably high alignment scores indicative of alignments with few mismatches/indels. If such read pairs exist it would argue against bisegmentation and rather suggest that a piece of the viral genome between the end of ORF1b and the most 5’ structural/accessory ORF was not sequenced due to some unknown reason. We did not identify any read pair for any of the analyzed viruses that fulfill the requirements described above, supporting our finding of genome segmentation of this subset of fish coronaviruses. We have added a description of and results from this new analysis to the manuscript.

Adding to the above, the following, mostly computationally derived points provide further convincing evidence for bisegmentation. Firstly, many of the assembled segment sequences have poly-A tails at their 3’-ends; for several viruses this is true for both segments (see main Fig. 4). Secondly, many segment 2 sequences have non-coding regions at their 5’-ends. For some viruses, Ambystoma mexicanum coronavirus is the prime example, we are confident that our assembly of both segments is virtually complete with poly-A tails at both 3’-ends and nearly complete 5’-UTRs. Thirdly, sequencing depth is very high for many of the segment sequences, for instance is the mean depth larger 1000 for both segments of Ambystoma mexicanum coronavirus, (see Supplementary Fig. 3) and also the two assembly quality metrics (meas and mico) show medium to high values for most of the segment sequences (see Supplementary Fig. 2). Lastly, we have already demonstrated that our approach allows for the discovery of authentic viruses in a pilot study (Lauber, Seitz et al., 2017, Cell Host Microbe 22:387). This study describes the discovery of nackednaviruses, a sister family to the hepadnaviruses, and involves extensive experimental characterization of some of the newly discovered viruses, showing that they are genuine.

**G3**: Since the first submission of our manuscript, discovery of some of the coronavirus genomes have been independently confirmed in another computational study using a similar approach (Edgar et al., 2022, Nature 602:142). Also, we updated our search for novel nidovirus sequences by analyses of new fish sequencing experiments that have been deposited to the SRA in the meantime. This resulted in the discovery of 14 additional novel vertebrate nidovirus genomes which include three bisegmented coronaviruses, using a protein sequence identity cut-off of <90% for distinguishing viruses. Altogether, 21 of the now 40 nidoviruses are reported for the first time in this study.

**G4**: As an extra quality control, we have revisited the assembling of the reported virus contigs. A human expert in viral genome assembly assessed the quality and completeness of the computationally derived contigs using conventional bioinformatics tools. Briefly, all sequencing reads of a library were aligned to the viral contig and a visual representation of this read alignment was inspected for possible assembly mistakes. Nucleotide Blast searches of the contig ends and well as of genome sequences of closely related viruses (if available) against the full set of sequencing reads of a library were used to identify possible reads that would extend the contig sequence or which could bridge regions of missing sequence. This was repeated until no further reads could be identified. This additional quality control resulted in the extension of several of the computationally derived contig sequence and led us to identify just a single assembly mistake within ORF1a of Hypomesus transpacificus coronavirus: it is in a poly-U stretch of a substantial fraction of sequencing reads of this genome region and results in a fortuitous frameshift which is corrected now. Therefore, this genome has the canonical ORF1a-ORF1b organization, and not the ORF1a1-ORF1a2-ORF1b organization as described in the previous version of the manuscript.

**3) Point-by-point response**

The concerns raised by the editor/reviewers and our response are in blue and black font color, respectively.

**Editor:**

E.1: All reviewers and editors agree that the paper presents highly interesting findings, in particular to the bisegmentation of newly described nidoviruses. However, in line with the saying that extraordinary claims require extraordinary evidence, all reviewers ask for very significant wet-experimental proof that these viruses indeed have bisegmented genomes.

Please see our general note G1 above.

**Reviewer #1:**

R1.1: This fascinating manuscript describes the discovery and genomic characterization of 25 novel nidoviruses using data from the Sequence Read Archive and a suite of clever bioinformatics approaches. The manuscript is highly detailed and written well and carefully. Although “mining” the SRA for novel viruses is common these days, the approaches used in this study are novel. They build upon previous work by the investigators and demonstrate the utility of the approach for answering questions about deep virus evolution. The resulting inferences about the evolution of the nidoviruses are very interesting and important for the field of virology. Overall, this manuscript is novel enough in its approaches, findings and conclusions that it should not be viewed in the same light as other “metagenomic virus discovery papers.” It’s really nice work.

We thank Dr. Goldberg for his very positive and encouraging evaluation of our study and for providing detailed feedback that helped to further improve our manuscript.

R1.2: One minor issue is that the paper is really about nidoviruses but the authors sometimes describe the study as being about coronaviruses (for example, the title). Of course, the whole world is interested in coronaviruses these days, but this paper is broader. I encourage the authors to consider not COVID-izing their findings, because the findings are important on their own.

We fully agree with the reviewer that the scope of our study goes beyond coronaviruses. We have modified the title and the main text at several relevant places including the Introduction and Discussion section.

R1.3: I like the MICO and MEAS approach. However, I can’t quite understand what was done at the bottom of page 6. More generally, are these statistics applicable across data sets and analyses, or are they specific to a particular analysis? It almost seems that the authors wish to introduce these metrics as an industry-standard, but again it’s not clear.

Yes, the MICO and MEAS metrics are applicable across data sets and we would love to see these or similar metrics to be adopted as community standards in the future. The mico and meas values (small letters) are the actual metric values calculated for an assembled sequence and are not readily interpretable and comparable across data sets. The MICO and MEAS values are the relative positions of the corresponding mico and meas in a reference population of mico and meas values. The statistical approach used is to partition the reference population into deciles (10 equally-sized bins) and then assigning a particular value to one of the deciles. That makes them easily comparable across data sets. We have revised the corresponding Results section to address these points.

R1.4: One set of items missing in this paper are details of the computer systems (physical and virtual) used to run the analyses, including statistics on such things as computational power and computational time. The sheer size of the database makes me suspect that these analyses took a lot of CPUs and a long time, but that’s not clear. Inclusion of this information would help clarify how generally accessible these methods are.

We have added a new section about the HPC system to the Methods section:

*“Computational resources*

Most of the computational analyses, in particular the Virushunter and Virusgatherer stages, were conducted on the high-performance computing system Taurus of the University of Technology (TU) Dresden consisting of >1800 computing nodes that provide around 60,000 CPU cores. This allowed us to analyze dozens to hundreds of datasets in parallel at a particular point in time, depending on cluster load. Between 24 to 128 CPUs were used for the analysis of an individual SRA experiment. The full analysis took several months for completion and was done in batches, which were defined by host groups (such as fishes, amphibians, mammals, etc) derived from the SRA metadata.”

R1.5: Throughout the manuscript, the authors state that they have found novel virus species and genera. Although their DEmARC approach is used by ICTV to help identify novel taxa, there are other considerations as well (e.g. genomic architecture beyond RDRP). I suggest that the reviewers qualify their findings by saying “putative taxa” or similar phrases, to indicate that the new viruses have not yet been formally assigned to taxa. I also hope the authors will submit a taxonomic proposal to ICTV.

We now refer to delineated viral taxa as operational taxonomic units (OTUs) in the case of genera and families or qualify our findings using "putative” or similar as suggested.

R1.6: Page 4. Is there evidence that the size of these databases is growing exponentially, or is the word “exponentially” used metaphorically to argue that these databases are growing in some non-linear fashion? If the rate of growth is indeed exponential, a reference might be in order. If not, consider using a less mathematically precise descriptor (or delete this phrase).

Changed to "with a non-linear rate".

R1.7: Page 4. Change “several novel coronavirus genera” to “several putatively novel coronavirus taxa” because these new viruses could be classified into other levels than genera (see above).

Changed as suggested.

R1.8: Page 5. The authors don’t present details of the iterative extension method in the Methods section. I believe I know what they’ve done because I do it myself. It’s a tedious process and somewhat subjective, but it works. I suspect that the ability of the authors to arrive at their final contigs depended on this process in some cases. If so, the method should be described in detail in, including any subjectivity involved (which is fine).

We have updated the relevant Methods section. See also our reply to comment R2.3 below. The "iterative extension method" is described in Alves et al. (2016), to which we refer in the Methods section.

R1.9: Page 6. It might be a good idea to qualify statements about taxonomy (see my comment above) by calling the 25 viruses “novel nidovirus taxa” or perhaps just “novel nidoviruses.”

Changed to "novel nidoviruses".

R1.10: Pages 6 and 7 and Figure 2. The difference between MICO and MEAS (capital letters) and mico and meas (small letters) isn’t clear. I think the former is for the population of sequences and the latter is for an individual sequence, but I found this difficult to understand.

See our reply to comment R1.3 above.

R1.11: Page 7. Change “we determined” to “we estimated” because the missing sequences could contain indels.

Changed as suggested.

R1.12: Page 7. Despite the DEmARC analysis (which is used for taxonomic classification) it might still be best to refer to the viruses as “19 putatively novel nidovirus genera” or something similar, to indicate that the taxonomy isn’t finalized.

Changed as suggested.

R1.13: Page 8. To readers not familiar with coronavirus genome architecture, it might not be clear why the bipartite ORF1 is striking. Perhaps change to “Unlike any described coronavirus of which we are aware, ORF1a of this virus…”

This sentence was about Hypomesus transpacificus coronavirus. Our extra assembly quality control led us to identify a single assembly mistake within ORF1a of this virus: it is in a poly-U stretch of a substantial fraction of sequencing reads of this genome region and results in a fortuitous frameshift which is corrected now. Therefore, this genome has the canonical ORF1a-ORF1b organization, and not the ORF1a1-ORF1a2-ORF1b organization as described in the previous version of the manuscript. This part of the text has been updated accordingly in the revised version of the manuscript.

R1.14: Page 8, end of first paragraph. How similar are the two consecutive ORFs to each other? This information might help support (or not) the interesting idea presented in the Discussion that this represents a duplication event. It’s also been hypothesized that the 3-4 ORFs immediately downstream of the replicase ORFs in the simian arteriviruses arose through a duplication event, so this might be worth mentioning. See Godeny, E.K., de Vries, A.A., Wang, X.C., Smith, S.L., and de Groot, R.J., Identification of the leader-body junctions for the viral subgenomic mRNAs and organization of the simian hemorrhagic fever virus genome: evidence for gene duplication during arterivirus evolution. J Virol, 1998. 72(1):862-7.

These two ORFs in the 36kb fish coronavirus genome (HTCV), both of which show significant similarity to S proteins of other coronaviruses, have a high degree of sequence divergence when compared to each other. They show a protein sequence identity of just 21% in the conserved part of the ORF (now mentioned in the Results). Therefore, the putative duplication event is ancient (e.g. happened a long time ago) or, alternatively, one of the ORFs was acquired from a heterologous source and not by gene duplication in a nidovirus ancestor. We have slightly expanded the discussion of this finding, including addition of the suggested reference (Godeny et al.).

R1.15: Page 8. The existence of bipartite coronavirus genomes is very interesting. Because this is such a novel finding, it might be worth reassuring readers that these genomes are not an artifact of the bioinformatic approach used to assemble the contigs. For example, if there were (for some unknown reason) a region of low/no coverage between the nonstructural and structural ORFs of a monopartite coronavirus genome, could the approach used erroneously produce two contigs, making it appear to be a bipartite genome? This is a “picky reviewer” point, I confess! But, this is very cool stuff, so I want to make sure that alternative explanations are considered/addressed.

This point is not at all "picky" but important. We consider it to be extremely unlikely that observed bisegmentation is due to sequencing or assembly artifacts. We consistently and exclusively observe these 2 disjoint contigs (aka segments) in 12 divergent members of a monophyletic viral lineage. The sequencing data sets in which we found the viruses have been produced by different laboratories. Furthermore, we extended our computational analysis to test the proposed scenario and added new data, including additional members of the coronavirus lineage with segmented genomes, further supporting our claim. For a more detailed reply please see our general notes G1and G2 above.

R1.16: Page 9. The discovery of glycosidase-like genes is very interesting. It would be worth alluding to the functions of these proteins in other viruses (e.g. influenza neuraminidase), to set things up for the Discussion on page 13.

Influenza virus neuraminidase is already mentioned as an example in this Results section. We prefer not to extend this list of viral glycosidase examples at this particular place in the manuscript.

R1.17: Page 10. “Zokor,” not “zoker.” “Zoker” sounds like a fossorial mammal that is the nemesis of Batman. :-)

Corrected as suggested.

R1.18: Page 10. Change to “(and a putative new genus).”

Changed as suggested.

R1.19: Page 10, last line. What is meant by “experiments?” Are these lab experiments where the animals were subject to experimental infection?

Changed to "sequencing experiments".

R1.20: Page 11. Consider deleting the sentences at the end of the first paragraph beginning with “In the light of these findings.” It wasn’t surprising even before these findings that SARS-CoV-2 was a recombinant, because homologous recombination between closely related coronaviruses has been known for a long time. Also, despite the importance of the COVID-19 pandemic, casting these results as being relevant to “pandemic preparedness” is a bit of a stretch. The relevance of these results is squarely in the realm of deep virus evolution, and the authors do a good job of stating that in the prior part of this paragraph. The quality of this paper stands on its own, pandemic notwithstanding!

We thank the reviewer again for his encouraging words. As suggested, we have deleted these last two sentences of the paragraph indicated by the reviewer.

R1.21: Sections on pages 14 and 15. I agree wholeheartedly about the advantages of mining the SRA for viruses and the associated caveats. The issue with these sections is that they are not directly related to the main virological findings of the study. And, they read as being somewhat defensive, as if anticipating criticism, and perhaps slightly self-congratulatory, which can be off-putting. I have no fundamental issue with their inclusion, but I am curious to see what the other reviewers say. It may also be an editorial decision. Regardless, I encourage the authors to consider writing a high-profile review paper about these issues. It would be a much-needed contribution to the field.

We appreciate the constructive feedback. We have slightly modified this part of the Discussion, which includes the addition of a perspective article on SRA-based virus discovery that we have published recently.

R1.22: Page 16. “Data” is plural (“SRA data were downloaded”). More picky reviewer stuff; sorry.

Corrected as suggested.

R1.23: Page 18. Is there a reference for LAMPA?

We are glad that the reviewer noticed our oversight and have now added a reference for LAMPA.

R1.24: Figure S6. The host taxon names are uninterpretable. What about writing the common name of the host and the accession number in parentheses? That’d allow the reader to appreciate the diversity of glycosidase genes across the vertebrates.

For reconstruction of this tree, we have used the GH18 seed alignment from Pfam and added the nidovirus sequences. We were unable to map the identifiers from the Pfam alignment to host names. We have included a comment as to how the interested reader could manually look up the host and other metadata information for certain sequence entries. Note that this tree includes the entire diversity of cellular organisms, not just vertebrates.

**Reviewer #2:**

R2.1: In their study the authors mined the Sequence Read Archive for novel nidoviruses. They detected 25 previously unknown sequences in different vertebrate species. The sequences grouped within the order *Nidovirales* in phylogenetic analyses. Interestingly, a great variety of unique genome organizations was found. For example, some of the detected viruses seemed to contain bipartite genomes, although all nidoviruses known to date contain single stranded positive sense RNA as genome. Furthermore, viruses with putatively duplicated spike proteins or divided ORF1a open reading frames were found. In addition, protein domains that have so far not been detected in nidoviruses were found.

The manuscript is very well written including a detailed introduction and discussion section providing all relevant information to understand and appreciate the findings of the authors. The methods are appropriate, and the conclusions supported by the data.

We thank this reviewer for the overall positive evaluation of our study and for providing feedback that helped to further improve our manuscript.

R2.2: Unfortunately, the result section is limited to the description and analysis of the detected genome sequences. Albeit this is done at great length, the result section appears to be a bit thin as no further experimental (and confirmatory) data are provided. I would like to see some experimental data that show that these bizarre genomes are indeed existing and functional in nature. For example, expression of spike proteins in vertebrate cells could be shown and further be analysed by western blotting, mass-spectrometry, or Edman sequencing etc. Or it could be shown that the divided ORF1a indeed leads to the production of a full length sgRNA of the length of ORF1a. Or the authors could have screened samples from the respective vertebrate host species for presence of their novel viruses and confirm that these genomes with doubled spike proteins, bipartite genomes or divided ORF1a truly exist in nature and are no artefact of false assembly or missing of next generation sequencing reads. In its current form, the manuscript may be better suited in a more computational journal.

We share the Reviewer concern about reproducibility and independent verification of main results to exclude fortuitous origins (see also R1.15). We thank the Reviewer for the suggestions toward this end. Experimental characterization will be considered at the next stage of this project for carefully selected representatives to ensure project feasibility and to maximize insight for the involved viruses, their hosts and the order *Nidovirales*. This project will require dedicated funding and virus-specific reagents that take time and resources to acquire. In the meantime, we addressed the raised concerns using various sequence-based analyses, including state-of-the art reported for the first time. We believe our initial and newly added results, from our group and published by others, satisfy these requirements. They validate main conclusions of this study beyond reasonable doubt. Specifically:

We have now added a 3’RACE-PCR analysis demonstrating the presence of poly-A tails at both segments for two of the fish coronaviruses with bisegmented genomes, StyCoV-1 and StyCoV-2, implying that these segments may function as mRNA as expected. We also added new data on the re-discovery of these two viruses, based on original and newly presented raw transcriptomics data of 69 fishes. These satisfy requirement of reproducibility. See also our general note G1 above.

To provide further evidence that the discovered viruses exist and are functional, we added new data about discontinuous transcription and the generation of 5’- and 3’- coterminal subgenomic RNAs for two of the discovered viruses (a tobanivirus and an arterivirus) to the manuscript (see new Fig. 6 and new subsection “Discontinuous transcription of newly discovered vertebrate nidoviruses”). Briefly, using sequence-based analyses, we identified reads spanning the leader-body-junction and looked at changes in sequencing read depth to determine the genomic position and sequence of putative transcriptional regulatory sequences (TRSs) and leaders which allowed us to infer the full-length subgenomic RNAs produced by these viruses for expression of the structural proteins. Sequencing read depth was found to be increasing steeply and step-wise towards the 3’-end of the viral genome. Body TRSs were identified upstream and in proximity to the start codon of each structural/accessory protein encoded downstream of ORF1b. Our findings are in full agreement with what has been shown for other arteri- and tobaniviruses.

On a more general note, we would like to stress that, given reliable genome assembling (see our replies to R1.15 and R2.3), the conducted comparative genomics analyses alone provide sufficient evidence that the discovered viral genomes “are indeed existing and functional in nature”. We observed strong similarities in genomic organization, gene content and order, and protein motif conservation of the newly described genomes with reference nidoviruses, which may not emerge by chance. We are not aware of any alternative explanations for existence of theses patterns other than through evolutionary divergence from a common ancestor, implying functionality.

R2.3: How did the authors control for correct assembly? How long is the minimal overlap for assembled reads / reads that align with contigs?

As described in the manuscript, assembly quality is assessed using two metrics (MEAS and MICO), via re-mapping of reads against the assembled sequences to quantify local and global sequencing depth (Supplementary Figs. 3,4,5), and by manual inspection. During iterative, seed-based (targeted) viral genome assembly, the ends of the current contig are cut and used as query to search for additional reads that align to the contigs ends. We used 45 nucleotides as the size of these cut contigs ends. The newly found reads are then used to extend the contig if they overlap with one of the contig ends by at least 20 nucleotides and with at least 85% sequence identity. We have now added this detailed information to the Methods section.

Since the time of the original manuscript submission, several coronaviruses have been independently rediscovered by another team using a different computational pipeline for the SRA deep mining (Edgar et al., 2022, Nature 602:142). The assembled genome sequences of two studies are almost identical, further supporting the pipeline design of this study.

R2.4: How were non-viral sequences reliably identified?

Most of the relevant information was already presented in the Methods section of the original manuscript, but we have now extended it further. Briefly, the reliable identification of nidovirus sequences, and thus the removal of non-viral sequences, involves several stages of the workflow. At the Virushunter step, a protein Blast (blastx) against the non-viral portion of RefSeq-Protein with a E-value cut-off of 1e-4 is used to identify and remove sequences that may give false positive hits and complicate genome assembly. After viral genome assembly at the virus assignment step, a profile HMM search using nidovirus 3CLpro, NiRAN, RdRp, ZBD, and HEL1 profiles as query and applying an E-value cut-off of 10 was used to identify candidate nidoviral sequences. A viral sequence was considered as belonging to the order *Nidovirales* either if it encoded the ordered array of protein domains 3CLpro-NiRAN-RdRp-ZBD-HEL1 conserved in nidoviruses or, in the case of partial sequences lacking some of these domains (but not the RdRp), if it grouped within the diversity of other nidoviruses in the RdRp phylogeny.

R2.5: Regarding the virus assignment stage, please indicate the cut-off level for including sequences in the subsequent analysis („we selected those that showed highest sequence similarity to known nidoviruses (virus assignment stage).“).

See our reply to R2.4 above.

R2.6: If available, it would be good to include information from which organs the viral sequences were derived („delineation of 25 novel nidovirus species with putative vertebrate hosts.“).

We have added a new supplementary table (table S1) specifying this information and some additional metadata to the manuscript. It can be seen from this table that the nidoviral sequences were derived from various organs. We have added this note to the Results section.

**Reviewer #3:**

R3.1: The manuscript by Lauber et al presents the results of the mining of the sequence read archive with “novel” computational tools for the detection of members of the Coronaviridae family that were previously missed by the original SRA submitters. Lauber et al claims the discovery of 25 novel nidovirus from a wide range of vertebrate hosts. Among the set, the authors describe 8 fish coronavirus with bipartite genomes, one virus with the giant coronavirus genome to date as well as a duplicated Spike glycoprotein. Additionally, some of these genomes are proposed to encode protein domains that have not been described for nidoviruses. The authors also posit they they provide evidence of an inter-family recombination between bipartite coronavirus and unsegmented tobaniviruses. The authors generate all this information purely with computational tools by re-analyzing publicly available datasets of data that were deposited in the Sequence Read Archive. The methodological approach involves a 3 stage approach that involved the initial detection of homology in the raw reads using Hidden Markov Models by a tool named “Virushunter”, and the extension of that initial seed by using the “Virusgatherer” tools until obtaining what the authors call “full-genome sequences”. Finally, virus assignment of the assembled tools would be performed by phylogenetic methods. While this process fundamentally does not differ from other pathogen discovery methods, the authors focus their research in the twilight zone (e.g. the area were protein similarities signal starts to fade in non-significance) and HMM-models, which allows them for the discovery of very distant viruses. The approach is of great interest. And it is clearly a novel path that must be explored and mined. It is clear that it is probably a method that could be applied to multiple different families, after identification of the appropriate Hidden Markov Model seeds.

We thank Dr. Palacios for his overall positive evaluation of our study and for providing detailed feedback that helped to further improve our manuscript.

R3.2: However, the value and significance of the product generated (e.g. the paper) is very difficult to assess and might need to define a new paradigm for publication.

We regret that the reviewer found the generated product difficult to assess. At the same time, we are pleased that this reviewer (see R3.1 above and R3.4 below) as well as another reviewer (see R1.1 above) both praised this study for novelty and advancement. We hope to resolve any remaining concerns of this reviewer in our revised version which includes new results generated by both computational and wet-lab analyses.

R3.3: As per the methodology, the approach is not completely novel, and even when the authors refer to their tools as new entities (e.g. virushunter and virusgatherer tools), according to the M&M, those bioinformatic pipeline do not exist as independent entities that can be downloaded and reproduced by others, but as a series of bioinformatic steps using multiple and redundant tools. OF note, the M&M section does not provide enough details for the independent analysis of the data. That should be solved before publication. In any event, there is not enough novelty in the bioinformatic approach alone to warrant publication in PLOS Pathogens.

We have extended the M&M section and made our tools available via github. As we detailed in reply to R1.4, the reported virus discoveries involved a state-of-the-art analysis at high performance computational facilities over several months. A virus discovery study using a similar approach was published by another group in *Nature* recently.

R3.4: On the other hand, their biological findings, if accepted as presented, would warrant publication, as they would be another clue of the importance of segmented genomes in viral evolution and they would confirm several of the more recent theories describing the virosphere. Then, IMHO, the impact and significance of this paper relies on the authors demonstrating unequivocally of the bipartite properties of the novel virus. Big claims require big proof.

We have now added new computational results as well as experimental data including a 3’RACE-PCR analysis demonstrating the presence of poly-A tails at both segments for two of the fish coronaviruses with bisegmented genomes. We also added new data on the re-discovery of these two viruses based on original and unpublished data of 69 fishes. See also our general notes G1-G3 above.

R3.5: Given that the authors are re-analyzing other peoples data, the Achilles heel of their approach is that they cannot “biologically” validate their approach, as they do not have access to the specimens. Although their analysis appear to be sound and solid, it still could be affected by multiple source of errors that are not in control of the authors. Contamination of sequencing projects is only one of them. But since evidence of contamination would force the authors to re-assess some of the claims of reassortment and bipartism, including measures to discard contamination as a source of error should be part of their experimental design. Merging datasets to reconstruct genomes does not help their case.

To gain access to specimens and exclude origin by contamination, we have teamed-up with the authors of the SRA data (the team of Dr. Olivia Roth; now co-authors of this manuscript) in which we identified two of the fish coronaviruses with bisegmented genomes, Syngnathus typhle coronavirus 1 (StyCoV-1) and Syngnathus typhle coronavirus 2 (StyCoV-2). For more details please refer to our general comment G1 above.

R3.6: The research team appears to be aware of this, and tried to develop new metrics of quality to assess this risk. Two novel metrics to measure the per-base (MICO) and contig quality accuracy of the reconstruction (MEAS). Unfortunately, those metrics only produce that in average, the quality of their reconstructed genomes appear to be similar (but lower) than the values obtained with a reference of RNA viruses assembly. The authors recognize that their value are lower, though; and that are even lower for the bipartite genomes. Given that, as stated above, this is the main claim of significance, this result does not help their case.

Yes, coverage/read depth and thus the assembly quality metrics are rather low for some of the discovered viruses, which is due to the underlying data of the studies that were not concerned with virus discovery and therefore did not attempt to, for instance, enrich for viral material prior to sequencing. However, there are also several of the discovered viruses, including bipartite coronaviruses, with very good read depth and thus quality values on par with commonly accepted in conventional virus genome assembling studies. For instance, the assembly of both segment sequences of Ambystoma mexicanum coronavirus is complete and of high quality. We also validated this via manual inspection of the assembly and read mapping. See also our general comment G2 above.

R3.7: The authors also state that they find differences in coverage between the different segments and they hypothesize how that could be biologically relevant. Why this is plausible, an alternate explanation could be that those pieces of the genome are coming from different biological entities (thus at different levels). Classical virology would request for the authors to demonstrate those pieces are packaged together.

We agree that characterization of viral particles would be a conventional way to establish that the observed two segments belong to the same genome. However, this analysis often involves tissue culturing and is beyond the realm of our study. At the same time, we believe that the provided data are sufficiently complete to argue against the alternative explanation raised by the reviewer. First, we only detected a single variant of each segment in a particular sample, in total 81 samples, fully in line with genome being bi-segmented. To consider that each of these segments pairs with another, non-observed segment, as the alternative hypothesis posits, this second segment must either be selectively degraded or produced in quantities below detection. These assumptions imply exotic properties for uncertain reasons that raise more questions than provide answers. Second, the division of labor in the nidovirus genome implies genes involved in replication and transcription compared to those involved in virus particles formation may require relatively few mRNA copies. This functionally driven mRNA allocation pattern was described for canonical non-segmented nidoviruses and was also observed for bi-segmented nidoviruses in this study: we identified replicative coronavirus proteins encoded on the less abundant segment 1 and proteins homologous to glycoproteins and nucleocapsid proteins of coronaviruses on the more abundant segment 2. Thus, we concluded that the two segments form the genome of a single coronavirus and that the observed differences in read depth between the two segments are biologically relevant.

R3.8: In addition, they DID not attempt to quantify genome completeness (defined as coverage of the complete protein coding part of the genome, 3’UTR or 5’UTR). (e.g. it Is a little confusing when the authors refer in the manuscript to “complete or nearly complete genome sequences” in other sections of the manuscript. That is a big problem in my view. Ensuring that the segmented bipartite viruses would be complete will provide evidence to the authors of their claims. Segmented viruses have developed strategies to pack different segment together. Segmented positive strand viruses should have similar structures for translation in their UTRs. Analysis of those regions for completeness and functionality would give the authors a significant quality booster to support their claims. It might not be necessary to demonstrate that all the virus are complete, but at least that some of the agents are. Moreover, as the majority of the SRA datasets meta information is well described, it is not clear why the authors did not attempt to test their finding in the wet-lab too.

As described above (see for instance our general comment G1), we have teamed-up with one of the submitters of the SRA data in which we discovered two bisegmented coronaviruses, enabling wet-lab testing.

Regarding genome (segment) completeness, we have performed an additional, extensive manual quality assessment and refinement of the assemblies. This resulted in the extension of many of our assembled nidovirus genomes. The applied method largely resembles our computational, seed-based assembly approach. Briefly, an expert in viral genome assembly (a new co-author on the manuscript who therefore provided an unbiased new view on the data) identified reads matching to the ends of a viral contig and aligns them to the contig. Putatively wrongly assembled parts of the computationally assembled sequence are corrected, if any are identified, and the contig is then iteratively extended using reads that partially overlap with the sequence termini. This strategy is repeated until no new viral reads are found. This manual approach allows to correct misassembled regions from the computationally derived contigs and to bridge regions of very low read coverage at which the computational assembly might terminate prematurely.

We are confident that many of our assemblies are virtually complete, including both segmented und unsegmented viruses. An example is given by the two Ambystoma mexicanum coronavirus (AmCoV) genome segments. Both segments end with a poly-A and show a 5'-UTR of sufficient length. We also consider it extremely unlikely that we have missed a (putative) third genome segment as all the expected structural and non-structural proteins are encoded on segment 2 and 1, respectively. Moreover, the 5'-ends of the two AmCoV segments and the 3'-ends of the two AmCoV segments show striking sequence similarity – 97.4% sequence identity across a stretch of 272 nt (positions 431-701 of segment 1 vs. positions 1-272 of segment 2) and 99.8% across 821 nt (positions 19446-20265 of segment 1 vs. positions 12489-13309 of segment 2), respectively. Similar observations have been made for other segmented RNA viruses (Qin et al., 2014, PNAS 111(18):6744-6749), suggesting that the two AmCoV segments are indeed packed together. We now specifically mention these data in the Results section.

R3.9: Finally, the question of significance still remains open. While I do not believe that the authors had completely ruled out all other possibilities of error in their viral genome reconstruction; I do believe that their explanation is probably correct. I do also believe that their work deserve publication and advance the field. Do we need a new category for “likely” in-silico only viral assemblies that would remain in that category until proper wet-lab methods could be applied? I believe this goes beyond the classical discussion between classical virologists (that only believe that a virus exist once it has been cultured in vivo or in vitro) and pathogen discoverers (that believe that in silicon sequences as proof enough of the existence of a virus entity).

We are pleased that, overall, the reviewer believes that our work deserves publication and advances the field. In our answers to the raised concerns, we showed that we addressed all plausible sources of technical errors or of biological nature that would otherwise compromise or complicate the main conclusions of this study. We observed very few single nucleotide polymorphisms in the assembled sequences. It indicates the lack of mixed infection of closely related but different viruses in the analyzed samples. Furthermore, almost identical genome sequences were recovered from several independently collected samples infected with StyCoV-1 and StyCoV-2. We conclude from these and other observations that we determined genome consensus sequences of respective newly discovered viruses. In the subsequent comparative sequence analyses, these sequences fit patterns of protein and nucleotide variation and conservation that were established in prior research of coronaviruses or, if they deviated, remained compatible with fundamentals of RNA virus evolution. Accordingly, the newly assembled genome sequences are as reliable as any other obtained in the course of conventional genomic characterization of viruses, which have informed virus research for several decades.

The present-day genome databases are dominated by sequences determined using NGS. These sequences are computationally assembled and were rarely validated in wet lab. The genome assembling pipeline used in our study could be considered as an advancement of the conventional NGS. Its use in a study could be specified, like any other NGS method, as a feature in GenBank entries of the respective sequences. Like conventional NGS, our pipeline relies upon a statistical evaluation of the genome sequence assembling quality using measures of read depth and coverage. The entire computational pipeline is now freely available and we hope that its use by others will facilitate its incorporation into tools for virus research.
