# Supplementary material for: Deep mining of the Sequence Read Archive reveals major genetic innovations in coronaviruses and other nidoviruses of aquatic vertebrates
Source: PLoS Pathog. 2024 Apr 22;20(4):e1012163. doi: 10.1371/journal.ppat.1012163 (PMC11065284; doi:10.1371/journal.ppat.1012163)
Supplement: Attachment — Submitted filename: PPATH_revision2_response_to_reviewer_comments.docx [file ppat.1012163.s011.docx]

*Response to reviewer comments to PPATHOGENS-D-23-01445R1 -“Deep mining of the Sequence Read Archive reveals major genetic innovations in coronaviruses and other nidoviruses of aquatic vertebrates”*

**Deep mining of the Sequence Read Archive reveals major genetic innovations in coronaviruses and other nidoviruses of aquatic vertebrates**

Chris Lauber, Xiaoyu Zhang, Josef Vaas, Franziska Klingler, Pascal Mutz, Arseny Dubin, Thomas Pietschmann, Olivia Roth, Benjamin W. Neuman, Alexander E. Gorbalenya, Ralf Bartenschlager, Stefan Seitz

**Point-by-point response**

The few remaining concerns raised by the editor/reviewers and our responses are in blue and black font color, respectively.

**Editor:**

The reviewers appreciated the attention to an important topic and agree that your team have significantly improved the work and thoroughly addressed all previous critiques. However, Reviewer 1 highlight that it is still important to further validate the existence of bi-segmented coronavirus genomes. Given that your team had access to available RNA samples positive for these bi-segmented genomes, one potentially straightforward approach would be to design a PCR strategy to assess if the amplification of a contiguous product is feasible. With the proper controls, the absence of an amplifiable contiguous product would provide additional evidence to support the existence of these bi-segmented genomes.

We thank the editor for the overall positive assessment of our revised manuscript. We have conducted the suggested PCR analysis and included it into the manuscript. Please see our response to Reviewer #1 below for details.

**Reviewer #1:**

The authors took great efforts to improve the manuscript and addressed the concerns of the reviewers. However, I am still surprised about the findings of bi-segmented coronaviruses. As a simple proof and since the authors now have RNA from infected fish available, did they try to combine segment 1 and 2 using specific primers and nested PCR?

We thank the reviewer for this comment. We have designed and applied the following nested PCR strategy for one of the bisegmented coronaviruses (Syngnathus typhle coronavirus 2, StyCoV-2) and included the results from this new analysis into the manuscript, resulting in an update of the Methods and Results sections and Supplementary Figure S5.

As control analyses, we designed outer and inner primer pairs for both segments of StyCoV-2. We chose StyCoV-2 because we consider both of its segment sequences to be coding complete, in contrast to those of StyCoV-1. The primer pairs for segment 1 align to a region near the end of the ORF1b coding region, e.g. close to the putative 3’-end of segment 1. The primer pairs for segment 2 align to a region near the start of the first ORF encoded by segment 2, e.g. close to the putative 5’-end of segment 2. We used the outer and inner primer pairs for nested RT-PCR and obtained amplification products of the expected size for both segments, confirming the presence of both segments in the sample and our ability to detect them using nested RT-PCR.

We then used the left (forward) outer and inner primers of segment 1 and the right (reverse) outer and inner primers of segmented 2 to perform another nested RT-PCR analysis trying to bridge the two segments (overgap PCR). This overgap PCR did not yield any amplification product, further supporting the presence of two segments, e.g. genome bisegmentation. A detailed scheme of the nested overgap and control PCR design is shown in Supplementary Figure S5D.

**Reviewer #2**

The paper by Lauber et al reports the discovery of bisegmented NIdoviruses by computational mining of SRA. This is a biologically interesting and important discovery, and in addition, the manuscript reports several methodological innovations that will be useful to increase the robustness of new virus discovery by mining single read databases. Furthermore, this revised version includes limited but convincing experimental validation of the bisegmented genome organization of the discovered viruses via the detection of polyA tail on both segments.

I find no major issues with the manuscript and believe that in this revision, the authors have more than adequately addressed all the concerns and suggestions of the reviewers. Further experiments would be beyond the scope of this work and should be pursued and published separately. The authors' response to the reviewers' comments is extremely thorough.

I see no issues with this manuscript, it is very well, clearly written.

We thank Dr. Koonin for reviewing our manuscript and we are glad that he did not identify any remaining issues.
